# Resistencia a la fatiga cíclica de las limas Protaper GOLD, 2Shape y X-Pro Gold con diferentes motores de uso endodóntico

**DOI:** 10.21142/2523-2754-1404-2026-316

**Published:** 2026-10-01

**Authors:** Fabiola del Pilar Matumay Santos, Carmen Rosa García-Rupaya

**Affiliations:** 1 Escuela de Estomatología, Universidad Científica del Sur. Lima, Perú. fmatumay@cientifica.edu.pe gcarmen_rosa@hotmail.com Universidad Científica del Sur Escuela de Estomatología Universidad Científica del Sur Lima Peru fmatumay@cientifica.edu.pe gcarmen_rosa@hotmail.com

**Keywords:** fatiga cíclica, endodoncia, instrumentos de níquel-titanio, cyclic fatigue, endodontics, nickel-titanium instruments

## Abstract

**Objetivo:**

Comparar la resistencia a la fatiga cíclica de limas NiTi ProTaper Gold, 2Shape y X-Pro Gold, activadas con motores VDW, X-Smart Plus y Motopex, en conducto simulado 90°.

**Materiales y métodos:**

Estudio *in vitro* con 90 limas NiTi, tres grupos por tipo, subdivididos por motor (n = 10 por combinación). Fatiga cíclica evaluada en platina de acero, conducto simulado 90°, radio 5 mm, diámetro 1,5 mm. Activación: 300 rpm y 3 N·cm hasta fractura. Se registraron los tiempos y ciclos hasta la fractura (NCF). Se aplicaron Anova factorial y Tukey *post hoc* (p < 0,05). Las superficies fueron examinadas con microscopía electrónica de barrido.

**Resultados:**

X-Pro Gold mostró significativamente mayor NCF que ProTaper Gold y 2Shape con todos los motores (p < 0,001). VDW: X-Pro Gold 1132 ± 165, mediana 1155, IC 95 % 1014-1572; ProTaper Gold 688 ± 131; 2Shape 250 ± 70. X-Smart Plus: X-Pro Gold 1264 ± 429, ProTaper Gold 928 ± 159; 2Shape 291 ± 71. Motopex: X-Pro Gold 917 ± 390, ProTaper Gold 765 ± 128; 2Shape 265 ± 46. Los resultados fueron consistentes y mostraron un desempeño superior de X-Pro Gold y menor fractura con todos los motores.

**Conclusión:**

La resistencia a la fatiga cíclica depende del diseño y el motor; X-Pro Gold presentó gran resistencia, lo que sugiere un menor riesgo de fractura durante la instrumentación clínica endodóntica.

## Abstract (en)

**Objective:**

To compare the cyclic fatigue resistance of NiTi rotary files ProTaper Gold, 2Shape, and X-Pro Gold activated with VDW, X-Smart Plus, and Motopex motors in a 90° simulated curved canal.

**Materials and methods:**

: An in vitro study with 90 NiTi files, three groups by type, subdivided by motor (n = 10 per combination). Cyclic fatigue was tested on a stainless-steel platform, simulated canal 90°, radius 5 mm, internal diameter 1.5 mm. Activation: 300 rpm and 3 N·cm until fracture. Time and number of cycles to fracture (NCF) were recorded. Factorial ANOVA and Tukey *post hoc* (p < 0.05). Fracture surfaces examined by scanning electron microscopy.

**Results:**

X-Pro Gold exhibited significantly higher NCF than ProTaper Gold and 2Shape with all motors (p < 0.001). VDW: X-Pro Gold 1132 ± 165, median 1155, 95 % CI 1014-1572; ProTaper Gold 688 ± 131; 2Shape 250 ± 70. X-Smart Plus: X-Pro Gold 1264 ± 429, ProTaper Gold 928 ± 159; 2Shape 291 ± 71. Motopex: X-Pro Gold 917 ± 390, ProTaper Gold 765 ± 128; 2Shape 265 ± 46. Results were consistent, showing superior performance of X-Pro Gold and lower fracture risk with all motors.

**Conclusion:**

Cyclic fatigue resistance depends on instrument design and motor; X-Pro Gold demonstrated robust resistance, suggesting reduced risk of fracture during clinical endodontic instrumentation.

## INTRODUCCIÓN

El uso de instrumentos rotatorios de níquel-titanio (NiTi) ha representado un avance sustancial en la preparación biomecánica de los conductos radiculares, debido a sus propiedades superiores de elasticidad, flexibilidad y eficiencia de corte, lo que permite una instrumentación más centrada y una reducción significativa de eventos iatrogénicos, como transportaciones, escalones y perforaciones radiculares ^1^^,^^2^. Estas características han contribuido a mejorar la predictibilidad clínica y a disminuir el tiempo operatorio en comparación con los instrumentos de acero inoxidable convencionales ^3^.

No obstante, a pesar de sus ventajas mecánicas, los instrumentos rotatorios de NiTi presentan como principal limitación la fractura inesperada durante la instrumentación, incluso en ausencia de deformaciones visibles previas, lo que representa un riesgo clínico relevante para el éxito del tratamiento endodóntico ^4^^,^^5^. Desde un punto de vista biomecánico, la fractura de estos instrumentos se asocia fundamentalmente a dos mecanismos: la fatiga cíclica y la sobrecarga torsional, siendo la primera responsable de aproximadamente el 70 % de las fracturas reportadas ^6^^,^^7^.

La fatiga cíclica ocurre cuando el instrumento rota repetidamente dentro de conductos curvos, lo que genera ciclos alternantes de tensión y compresión en la zona de máxima curvatura que conducen a la iniciación y propagación progresiva de microgrietas hasta la falla del material ^8^^,^^9^. Factores como el diámetro del instrumento, la conicidad, el diseño de la sección transversal y la microestructura de la aleación influyen directamente en la resistencia a la fatiga cíclica, de modo que instrumentos con mayor diámetro o rigidez presentan, en general, menor resistencia a este tipo de carga mecánica ^10^^,^^11^.

Con el objetivo de mejorar el desempeño mecánico y reducir la incidencia de fracturas, se han desarrollado nuevos sistemas de instrumentación rotatoria mediante modificaciones en el diseño geométrico y la aplicación de tratamientos térmicos y procesos termomecánicos, los cuales alteran la microestructura del NiTi, favorecen la fase martensítica y optimizan su comportamiento elástico y su resistencia a la fatiga ^12^^-^^14^. En este contexto, sistemas contemporáneos como ProTaper Gold, 2Shape y X-Pro Gold incorporan aleaciones tratadas térmicamente y configuraciones específicas de sección transversal orientadas a equilibrar flexibilidad, capacidad de corte y resistencia mecánica ^15^^-^^17^.

Sin embargo, el comportamiento mecánico de las limas NiTi no depende exclusivamente de sus propiedades intrínsecas. La cinemática de rotación y el motor endodóntico empleado constituyen variables determinantes en la generación y distribución de las tensiones mecánicas durante la instrumentación ^18^^,^^19^. Los motores endodónticos modernos permiten el control preciso de parámetros como velocidad, torque, inversión automática y tipo de movimiento, factores que han demostrado influir significativamente en la resistencia a la fatiga cíclica y en la vida útil de los instrumentos rotatorios ^20^^-^^22^.

A pesar de la evidencia existente sobre las propiedades individuales de las limas NiTi y de los sistemas motorizados, son limitados los estudios experimentales que evalúan de manera conjunta el efecto del tipo de instrumento y del motor endodóntico mediante diseños factoriales, particularmente en condiciones de curvatura severa ^24^^,^^25^. En este contexto, la presente investigación tuvo como objetivo comparar la resistencia a la fatiga cíclica de las limas rotatorias ProTaper Gold, 2Shape y X-Pro Gold, activadas mediante diferentes motores de uso endodóntico, utilizando un modelo de conducto simulado con curvatura de 90°, con el fin de aportar evidencia experimental que permita comprender el efecto combinado de ambas variables sobre el comportamiento mecánico de los instrumentos.

## MATERIALES Y MÉTODOS

### Diseño del estudio

Se llevó a cabo un estudio experimental *in vitro* con el objetivo de evaluar la resistencia a la fatiga cíclica de limas rotatorias de níquel-titanio (NiTi) con tratamiento térmico, activadas mediante diferentes motores endodónticos. Aunque se trata de un estudio *in vitro*, el protocolo fue evaluado y aprobado por el Comité Institucional de Ética en Investigación de la Universidad Científica del Sur (número de aprobación: N.° 294-2021-POS8).

### Muestra y grupos experimentales

La muestra estuvo constituida por 90 limas rotatorias de NiTi, distribuidas en tres grupos según el sistema de instrumentación: grupo control (C1) ProTaper Gold F2 (Dentsply Maillefer, Suiza), grupo experimental 1 (G1) 2Shape TS2 (Micro-Mega, Besançon, Francia) y grupo experimental 2 (G2) X-Pro Gold F2 (Easyinsmile, Andorra). Cada grupo estuvo conformado por 30 limas.

Todas las limas presentaron un diámetro apical de 0,25 mm y una longitud de 25 mm. Cada grupo fue subdividido en tres subgrupos (n = 10), según el motor endodóntico utilizado: X-Smart Plus (Dentsply Sirona, número de catálogo A1033, Ballaigues, Suiza), Reciproc Silver (VDW, número de catálogo V04116300000, Múnich, Alemania) y Motopex (Woodpecker, número de catálogo C2000212UP, Guangxi, China).

### Criterios de selección

Se incluyeron limas de NiTi con tratamiento térmico, nuevas y con empaque sellado. Se excluyeron aquellas que presentaron defectos estructurales o deformaciones visibles al ser inspeccionadas bajo magnificación de 100× mediante un estereomicroscopio digital equipado con un sistema de captura de imágenes Digital Sight DS-F2 (Nikon Corporation, Tokio, Japón; serie Digital Sight).

Durante esta inspección inicial, se identificaron tres limas del grupo X-Pro Gold con microfisuras y bordes cortantes irregulares, las cuales fueron descartadas y reemplazadas por instrumentos de la misma marca sin defectos de fabricación (Figura 1).

**Figura 1:**
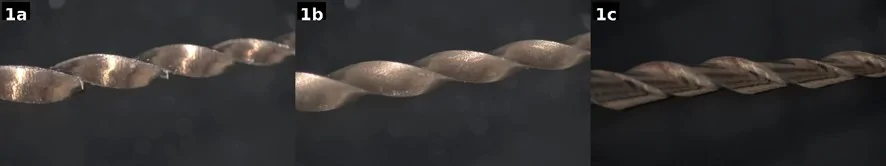
Imágenes representativas de los sistemas de limas endodónticas evaluadas mediante estereomicroscopía a una magnificación de 100X: 1a) ProTaper Gold (Dentsply Sirona, Ballaigues, Suiza); 1b) X-Pro Gold (FKG Dentaire, La Chaux-de-Fonds, Suiza); 1c) 2Shape (Micro-Mega, Besançon, Francia).

### Fabricación del modelo del conducto simulado

Se confeccionó un modelo de conducto simulado en una platina de acero inoxidable de 7 × 6 cm, diseñada para resistir el desgaste por fricción. El conducto presentó una curvatura de 90°, un radio de curvatura de 5 mm, un diámetro interno de 1,5 mm y una longitud total de 20 mm, distribuida en tres segmentos: uno inicial recto de 8 mm, un segmento curvo de 4 mm y un segmento final recto de 8 mm.

La platina fue cubierta en su cara anterior con un visor de acrílico transparente de 4 mm de espesor, lo que permitió visualizar el momento de la fractura y evitar el desplazamiento del instrumento durante la prueba.

### Prueba de resistencia a la fatiga cíclica

Los motores endodónticos utilizados fueron X-Smart Plus (Dentsply Sirona, Ballaigues, Suiza; número de catálogo: A1033), Reciproc Silver (VDW, Múnich, Alemania; número de catálogo: V04116300000) y Motopex (Woodpecker, Guangxi, China; número de catálogo: C2000212UP). Cada uno fue fijado en una posición reproducible mediante un soporte estandarizado (Figura 2). Las limas fueron introducidas en el conducto simulado hasta una longitud de trabajo de 18 mm, lo cual se mantuvo constante para todos los grupos (Figura 2).

**Figura 2:**
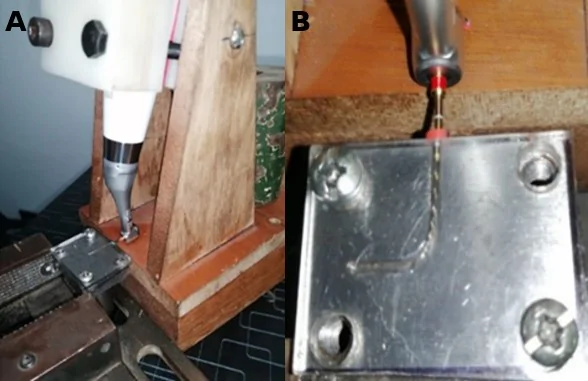
Dispositivo experimental utilizado para la evaluación de la resistencia a la fatiga cíclica. A) Soporte diseñado para mantener el contraángulo en posición reproducible. B) Medición de la lima a 18 mm para su inserción en un conducto artificial con curvatura de 90°.

Los instrumentos se activaron en rotación continua, utilizando las revoluciones por minuto y el torque recomendados por el fabricante (300 rpm y 3 N·cm). El tiempo hasta la fractura fue registrado en segundos mediante un cronómetro digital, lo cual permitió que las limas giraran libremente dentro del conducto hasta su fractura.

Con el fin de reducir la fricción entre el instrumento y la platina de acero inoxidable, se aplicó un lubricante sintético para piezas mecánicas (TS-1140) antes de cada prueba. Asimismo, los motores endodónticos fueron recalibrados tras el uso de cada instrumento para minimizar errores derivados de la mecánica del equipo.

El número de ciclos hasta la fractura (NCF) se calculó mediante la siguiente fórmula: NCF = (300 rpm × tiempo hasta la fractura en segundos) / 60.

### Medición del fragmento fracturado

La longitud de los segmentos fracturados fue medida en milímetros utilizando un calibrador vernier digital (Mitutoyo Corporation, número de catálogo: 500-196-3, Japón), por un ingeniero capacitado. Los datos correspondientes al tiempo hasta la fractura y a la longitud de los fragmentos fueron consignados en un informe técnico emitido por High Technology Laboratory Certificate S. A. C. (ensayo IE-0254-2022).

### Análisis estadístico

Los datos obtenidos fueron procesados mediante el programa SPSS Statistics versión 27 (IBM, Armonk, NY, EE. UU.). Se realizó un análisis estadístico descriptivo con el fin de calcular la media, la desviación estándar, la mediana y el intervalo de confianza al 95% del número de ciclos hasta la fractura (NCF), para cada combinación lima-motor.

Previo al análisis inferencial, se evaluaron los supuestos de normalidad de los datos. Posteriormente, el efecto de las variables experimentales sobre el NCF se analizó mediante un modelo lineal general (GLM), utilizando un análisis de varianza factorial, en el que se consideró la combinación lima-motor como factor fijo con nueve niveles experimentales (tres tipos de limas y tres motores endodónticos).

El análisis inferencial se realizó empleando sumas de cuadrados tipo III y se obtuvo los valores de suma de cuadrados, grados de libertad, media cuadrática, estadístico F y nivel de significancia (p). Asimismo, se estimó el coeficiente de determinación (R²) y su valor ajustado, con el fin de cuantificar la proporción de la variabilidad total del NCF explicada por el modelo. Cuando se identificaron diferencias estadísticamente significativas, se aplicaron pruebas *post hoc* de comparaciones múltiples de Tukey, y se estableció un nivel de significancia del 5 % (p < 0,05).

### Análisis mediante microscopía electrónica de barrido

El análisis fractográfico mediante microscopía electrónica de barrido (SEM) se realizó en el Laboratorio de Equipamiento Especializado de la Facultad de Ciencias Biológicas de la Universidad Nacional Mayor de San Marcos. Se seleccionaron de manera aleatoria las superficies fracturadas de los tres sistemas de limas de níquel-titanio evaluados (ProTaper Gold, 2Shape y X-Pro Gold), activados con los tres motores endodónticos incluidos en el estudio (X-Smart Plus, Silver Reciproc VDW y Motopex), a fin de caracterizar el modo de falla asociado con la fractura de los instrumentos.

Las observaciones se realizaron mediante microscopía electrónica de barrido (FEI Inspect S50, FEI Company, Hillsboro, OR, EE. UU.) y se obtuvo microfotografías a aumentos de 600×, 2000× y 3000×. Estos aumentos permitieron evaluar en detalle la topografía de las superficies fracturadas y la presencia de características fractográficas compatibles con mecanismos de fatiga cíclica.

El análisis mediante SEM correspondió a una evaluación cualitativa de tipo descriptivo y no cuantitativa, orientada a la identificación de zonas de iniciación de grietas, áreas de propagación progresiva de la fractura, bordes desgastados y hoyuelos, parámetros morfológicos comúnmente asociados a fallas por fatiga cíclica en instrumentos rotatorios de NiTi, de acuerdo con criterios previamente descritos en la literatura ^26^^,^^27^.

## RESULTADOS

Se evaluó la resistencia a la fatiga cíclica de limas ProTaper Gold, 2Shape y X-Pro Gold utilizando diferentes motores endodónticos, mediante el número de ciclos hasta la fractura (NCF). Los resultados evidenciaron diferencias en el desempeño mecánico de las limas según el tipo de instrumento y el motor empleado, y se observó, en términos generales, mayores valores de NCF en las limas X-Pro Gold y menores valores en las limas 2Shape, con variaciones dependientes de la combinación evaluada.

La estadística descriptiva de los valores de NCF para cada combinación lima-motor se presenta en la Tabla 1, donde se aprecia una dispersión variable de los datos y diferencias en las medidas de tendencia central entre los grupos analizados.

**Tabla 1:** Número de ciclos hasta la fractura (NCF) según el tipo de lima (ProTaper Gold, 2Shape y X-Pro Gold) y el motor endodóntico empleado

Tipo de motor	Tipo de lima	n	NCF		
			Media (DS)	Mediana	IC
VDW	ProTaper Gold	10	687,57 (131,43)	710,1	593,55-781,58
	2Shape	10	250,17 (70,07)	235,2	200,05-300,29
	X-Pro Gold	10	1132,02 (164,83)	1155	1014,11-1571,62
X-Smart	ProTaper	10	927,54 (159,41)	935,1	813,50-1041,58
	2Shape	10	291,06 (70,97)	269,85	240,29-341,83
	X-Pro Gold	10	1264,41 (429,45)	1320	957,19-1571,62
Motopex	ProTaper Gold	10	765.45 (128.139)	710,1	673,61-857,29
	2Shape	10	265,03 (45,91)	260,18	232,18-297,87
	X-Pro Gold	10	917,07 (390.16)	812,55	637,97-1196,17

Nota: Los valores se expresan como media ± desviación estándar (DE), mediana e intervalo de confianza al 95 % (IC 95 %). Cada grupo incluyó n = 10 instrumentos. El análisis inferencial se realizó considerando la combinación lima-motor como unidad experimental.

El análisis factorial confirmó un efecto estadísticamente significativo de la combinación lima-motor sobre el número de ciclos hasta la fractura (F = 30,07; gl = 8,81; p < 0,001), lo que explica el 74,8 % de la variabilidad total del NCF (R² = 0,748) (Tabla 2).

**Tabla 2:** Análisis factorial del efecto de la combinación lima-motor sobre los ciclos hasta la fractura (NCF)

Fuente de variación	Suma de cuadrados (Tipo III)	gl	Media cuadrática	F	p
Lima-motor	11 628 520,62	8	1 453 565,08	30,07	< 0,001
Error	3 915 397,04	81	48 338,24		
Total corregido	15 543 917,66	89			

Nota: Se realizó un análisis de varianza considerando la combinación lima-motor como factor fijo (9 niveles). gl: grados de libertad; F: estadístico F de Fisher; p: nivel de significancia. R² = 0,748 (R² ajustado=0,723).

El análisis fractográfico mediante microscopía electrónica de barrido (SEM) evidenció características compatibles con mecanismos de fatiga cíclica en todos los sistemas de limas evaluados (ProTaper Gold, X-Pro Gold y 2Shape) (Figuras 3-5).

**Figura 3:**
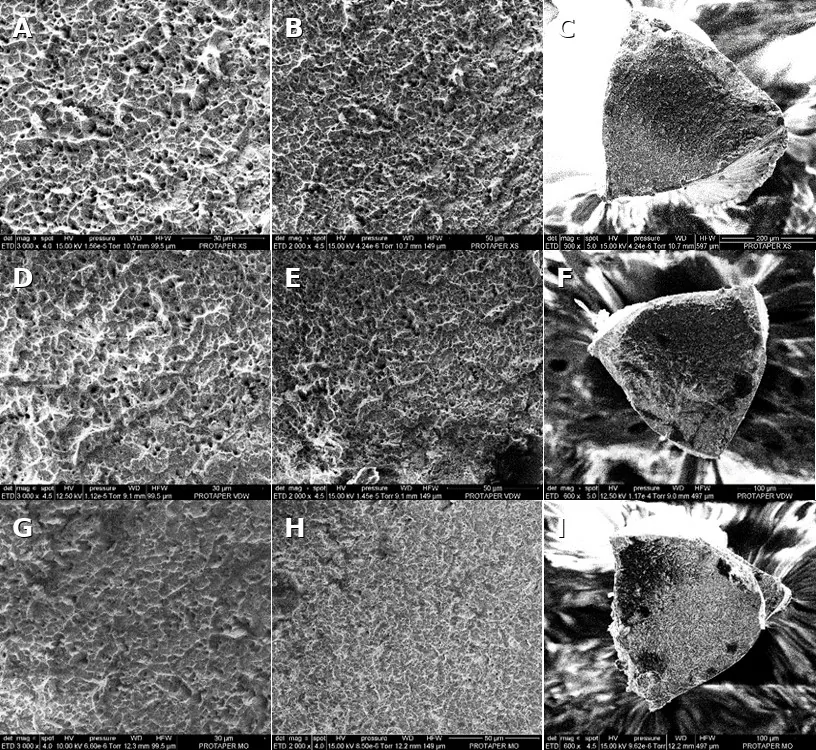
Micrografías obtenidas mediante microscopía electrónica de barrido (SEM) de las superficies de fractura de limas rotatorias ProTaper Gold tras ensayos de fatiga cíclica con diferentes motores endodónticos. A-C) Grupo X-Smart Plus: vista general (A), mayor aumento con evidencias de propagación de grietas (B) y zona final de fractura (C). D-F) Grupo VDW: vista general (D), detalle de la microestructura con patrón de propagación (E) y región final con características de fractura dúctil (F). G-I) Grupo Motopex: vista general (G), superficie con patrón homogéneo de fatiga (H) y zona final de fractura con presencia de dimples (I). En todos los grupos se observan características compatibles con fractura por fatiga cíclica.

**Figura 4:**
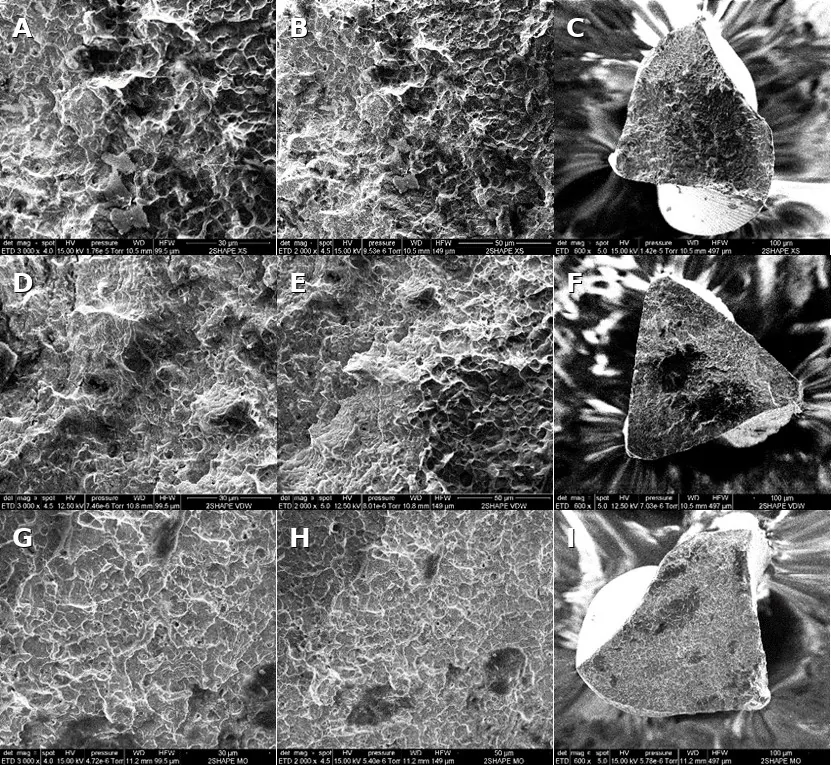
Micrografías obtenidas mediante microscopía electrónica de barrido (SEM) de las superficies de fractura de limas rotatorias X-Pro Gold tras ensayos de fatiga cíclica con diferentes motores endodónticos. A-C) Grupo X-Smart Plus: vista general (A), mayor aumento con evidencias de propagación de grietas (B) y zona final de fractura (C). D-F) Grupo VDW: vista general (D), detalle de la microestructura con patrón de propagación (E) y región final con características de fractura dúctil (F). G-I) Grupo Motopex: vista general (G), superficie con patrón homogéneo de fatiga (H) y zona final de fractura con presencia de dimples (I). Se observan patrones similares de fractura por fatiga cíclica en todos los grupos.

**Figura 5:**
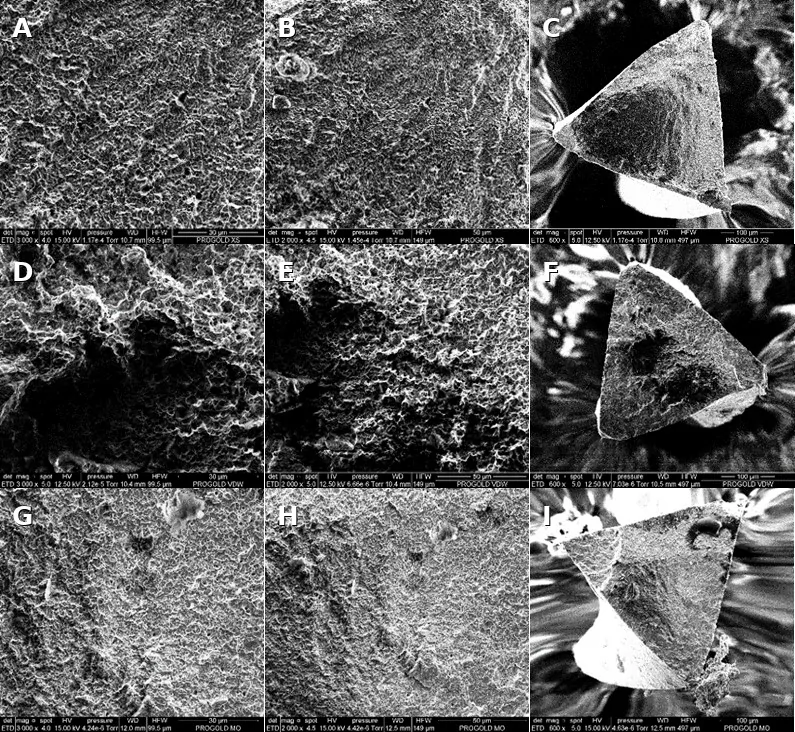
Micrografías obtenidas mediante microscopía electrónica de barrido (SEM) de las superficies de fractura de limas rotatorias 2Shape tras ensayos de fatiga cíclica con diferentes motores endodónticos. A-C) Grupo X-Smart Plus: vista general (A), mayor aumento con evidencias de propagación de grietas (B) y zona final de fractura (C). D-F) Grupo VDW: vista general (D), detalle de la microestructura con patrón de propagación (E) y región final con características de fractura dúctil (F). G-I) Grupo Motopex: vista general (G), superficie con patrón homogéneo de fatiga (H) y zona final de fractura con presencia de dimples (I). Se evidencian características comparables de fractura por fatiga cíclica entre los grupos evaluados.

En las micrografías de baja magnificación se observó una superficie de fractura relativamente plana, con un punto de inicio de la grieta localizado en la zona de máxima curvatura, como se aprecia en las vistas generales (Figuras 3A, 4A y 5A). A mayores aumentos, se identificaron estriaciones paralelas indicativas de la propagación progresiva de la grieta (Figuras 3B, 4B y 5B).

En la región final de fractura se evidenciaron superficies irregulares con morfología tipo dimples, compatibles con un mecanismo de fractura dúctil en la fase terminal (Figuras 3C, 4C y 5C). Asimismo, se observaron bordes irregulares y zonas de deformación plástica en algunos especímenes.

No se identificaron patrones predominantes de fractura torsional, tales como deformación helicoidal marcada o líneas concéntricas, lo cual sugiere que el mecanismo principal de falla en todos los grupos fue la fatiga cíclica.

Al comparar los diferentes sistemas de limas, los patrones fractográficos fueron similares entre ProTaper Gold (Figura 3), X-Pro Gold (Figura 4) y 2Shape (Figura 5), con ligeras variaciones en la extensión de las zonas de propagación de grietas.

## DISCUSIÓN

La resistencia a la fatiga cíclica, expresada como número de ciclos hasta la fractura (NCF), constituye un parámetro mecánico fundamental para evaluar el desempeño y la seguridad clínica de los instrumentos endodónticos de níquel-titanio, especialmente en conductos radiculares con curvaturas severas. En el presente estudio, el análisis factorial evidenció un efecto estadísticamente significativo de la combinación lima-motor sobre el NCF (F = 30,07; p < 0,001), lo que explica el 74,8 % de la variabilidad total observada. Esto confirma que el comportamiento frente a la fatiga cíclica está condicionado tanto por las características del instrumento como por el sistema motorizado empleado.

En términos comparativos, las limas X-Pro Gold mostraron consistentemente los mayores valores de NCF en los tres motores evaluados, y alcanzaron medias superiores a 1100 ciclos con los motores VDW y X-Smart Plus. Estos resultados concuerdan con lo reportado por Savitha *et al*. ^28^ y Tanomaru-Filho *et al*. ^29^, quienes demostraron que los instrumentos fabricados con aleaciones de NiTi sometidas a tratamientos térmicos avanzados presentan una mayor resistencia a la fatiga cíclica, atribuida a una mayor flexibilidad y capacidad de deformación reversible. De manera concordante, Braga *et al*. ^30^ y Zupanc *et al*. ^15^ señalaron que el control de la transformación de fases en estas aleaciones influye directamente en la vida a fatiga, lo que respalda el desempeño observado en las limas X-Pro Gold.

Por el contrario, las limas 2Shape presentaron los valores más bajos de NCF en todas las combinaciones evaluadas, con medias cercanas a los 250-290 ciclos, independientemente del motor utilizado. Estos hallazgos son consistentes con estudios previos que reportaron una menor resistencia a la fatiga cíclica para este sistema en comparación con otros instrumentos tratados térmicamente ^17^^,^^31^^,^^32^. Esta menor vida ante la fatiga podría explicarse por diferencias en el diseño transversal y la masa metálica, factores que influyen directamente en la concentración de tensiones en la zona de máxima curvatura del conducto, tal como fue señalado por Mehra *et al*. ^21^ y Faus-Llácer *et al*. ^33^.

Las limas ProTaper Gold mostraron valores intermedios de NCF, con variaciones dependientes del motor empleado. En particular, el mayor NCF observado con el motor X-Smart Plus sugiere que el control de la velocidad, el torque y la estabilidad cinemática pueden influir en la acumulación de tensiones cíclicas. Estudios previos han demostrado que modificaciones en la cinemática de rotación y en los parámetros operativos del motor pueden alterar significativamente la resistencia a la fatiga cíclica de un mismo instrumento ^21^^,^^22^^,^^34^.

Desde una perspectiva biomecánica, la fatiga cíclica se produce como consecuencia de la aplicación repetitiva de tensiones alternantes de compresión y tracción en la zona de máxima curvatura del conducto radicular, lo que conduce a la iniciación y propagación progresiva de microgrietas hasta ocasionar una fractura súbita del instrumento sin deformación plástica previa. La velocidad de propagación de estas microgrietas depende de la microestructura del NiTi y del diseño geométrico del instrumento, siendo menor en aquellos sistemas con mayor flexibilidad estructural ^35^^,^^36^.

En este contexto, el análisis fractográfico mediante microscopía electrónica de barrido (SEM) aportó evidencia complementaria para la interpretación de los resultados mecánicos. Este método ha sido ampliamente utilizado para evaluar el origen y la propagación de las grietas responsables de la falla de instrumentos de NiTi, lo que ha permitido diferenciar patrones asociados a fatiga cíclica y falla torsional ^35^. En el presente estudio, las superficies de fractura de las tres limas evaluadas presentaron características fractográficas similares, compatibles con un comportamiento típico de fatiga cíclica de naturaleza dúctil, tales como zonas de iniciación de grietas, bordes desgastados y morfología tipo copa-cono, sin evidenciar signos característicos de falla torsional. Estos hallazgos confirman que la fractura observada fue consecuencia de la acumulación progresiva de daño por cargas cíclicas y no de una sobrecarga torsional puntual.

El análisis fractográfico mediante SEM permitió identificar, en todos los grupos evaluados, zonas localizadas de iniciación de grietas y superficies de fractura con patrón rugoso y microcavitaciones, hallazgos característicos de un comportamiento dúctil asociado a fatiga cíclica. La ausencia de deformación plástica previa, estriaciones helicoidales o signos de sobrecarga torsional refuerza que la fractura ocurrió como consecuencia de tensiones alternantes de flexión repetida, más que por un fallo torsional abrupto. Este patrón fractográfico homogéneo entre las diferentes limas y motores coincide con lo reportado por Pedullà *et al*. ^24^, Shim *et al*. ^35^ y Gavini *et al*. ^37^, quienes describen que los instrumentos de NiTi sometidos a fatiga cíclica presentan mecanismos de falla similares, independientemente de las diferencias en diseño geométrico o tratamiento térmico.

El aporte principal del presente estudio radica en la evaluación conjunta del tipo de lima y del motor endodóntico como unidad experimental, complementada con el análisis fractográfico por SEM. Este enfoque integral permitió correlacionar los valores cuantitativos de resistencia a la fatiga cíclica con la morfología de las superficies de fractura, y proporciona así una comprensión más profunda del comportamiento mecánico de las limas durante la preparación biomecánica en conductos simulados con una curvatura severa de 90°. En este sentido, el mayor NCF observado en el grupo X-Pro Gold activado con el motor X-Smart Plus sugiere una interacción favorable entre las características del instrumento y la cinemática del motor.

Entre las limitaciones del presente estudio debe considerarse su diseño in vitro, el cual no reproduce completamente las condiciones clínicas reales, tales como la variabilidad anatómica del sistema de conductos radiculares, la presencia de irrigantes ni la influencia del operador durante la instrumentación. Asimismo, el uso de conductos artificiales con curvatura estandarizada pudo haber influido en los valores absolutos del número de ciclos hasta la fractura (NCF). No obstante, este tipo de metodología continúa siendo ampliamente aceptada para la comparación objetiva del comportamiento mecánico de instrumentos endodónticos, siempre que los resultados sean interpretados dentro de su contexto experimental ^14^^,^^37^.

Adicionalmente, los ensayos de fatiga cíclica fueron realizados a temperatura ambiente, sin control térmico, lo cual constituye una limitación relevante, considerando que se ha demostrado que la temperatura corporal puede modificar el comportamiento mecánico de las aleaciones de níquel-titanio mediante cambios en su fase cristalina, con una consecuente reducción de la resistencia a la fatiga cíclica. En este sentido, los valores absolutos de NCF obtenidos no deben extrapolarse directamente a condiciones clínicas. Sin embargo, esta limitación no afecta la validez de las comparaciones internas entre los grupos, dado que todas las limas fueron evaluadas bajo condiciones experimentales idénticas, lo que permitió un análisis comparativo consistente del efecto de la combinación lima-motor.

Finalmente, si bien el tamaño muestral fue establecido con base en estudios previos de fatiga cíclica con diseños experimentales similares, las investigaciones futuras deberían incorporar ensayos a temperatura corporal, análisis de potencia estadística y la evaluación de otras propiedades físicas y mecánicas de las limas, así como profundizar en la caracterización fractográfica de distintos segmentos del instrumento bajo diferentes sistemas motorizados, con el fin de ampliar la comprensión de su comportamiento clínico.

## CONCLUSIONES

Los resultados de este estudio *in vitro* indican que la resistencia a la fatiga cíclica de las limas rotatorias de níquel-titanio está influenciada por la combinación entre el tipo de instrumento y el motor endodóntico empleado, y se evidencia un efecto significativo de esta interacción sobre el número de ciclos hasta la fractura.

Las limas X-Pro Gold presentaron, en general, mayores valores de resistencia a la fatiga cíclica, seguidas por ProTaper Gold, mientras que el sistema 2Shape mostró los valores más bajos, independientemente del motor utilizado. Asimismo, se observaron diferencias en el patrón de fractura, con segmentos más largos en el sistema 2Shape cuando fue activado con el motor Motopex.

La identificación de defectos de fabricación en algunas limas X-Pro Gold resalta la importancia de considerar la calidad del instrumento como un factor adicional que puede influir en su comportamiento mecánico.

En conjunto, estos hallazgos sugieren que la selección clínica de los sistemas rotatorios NiTi debe considerar tanto el tipo de lima como el motor endodóntico, a fin de optimizar la seguridad y durabilidad durante la preparación biomecánica de conductos curvos, si bien se reconocen las limitaciones inherentes a un diseño experimental *in vitro.*

## Fuente de financiamiento:

El presente estudio fue financiado íntegramente por la autora principal, sin participación de entidades externas en el diseño del estudio, recolección de datos, análisis o redacción del manuscrito.
